# Use of Novel Blunt Dissection Technique for Surgical Unroofing in Myocardial Bridging Patients

**DOI:** 10.1155/2022/2370802

**Published:** 2022-11-09

**Authors:** Xiangyang Wu, Jie Zhu, Yong Mao, Xinqiang Guan, Xiaofeng He, Qi Ma, Xiaopeng Zhang, Shixiong Wang, Yongnan Li

**Affiliations:** Department of Cardiac Surgery, Lanzhou University Second Hospital, Lanzhou University, Lanzhou, China

## Abstract

**Background:**

Myocardial bridging (MB) is a congenital anomaly involving the myocardial tissue encasement of a segment of the coronary artery. The purpose of the present study was to assess safety and efficacy of two surgical methods used for treating MB patients at our institute.

**Methods:**

Off-pump MB unroofing was performed in 45 adult patients between January 2016 and December 2021 by traditional surgical unroofing techniques (conventional group, *n* = 26) and blunt dissection techniques (blunt dissection group, *n* = 19). We retrospectively reviewed our patients by examining the baseline clinical characteristics, risk factors, medications, and diagnostic data for coronary artery disease. The Seattle Angina Questionnaire (SAQ) was used to assess angina symptoms both preoperatively and 6 months postsurgery.

**Results:**

No significant difference in preoperative clinical characteristics was observed between the two groups. The blunt dissection group had shorter unroofed period (14.69 vs. 18.91 mins, *P*=0.001), less ventilator time (16.26 vs. 24.62 hours, *P* < 0.001), and a shorter hospital stay (8.74 vs. 12.89 days, *P* < 0.001). Although both traditional and blunt dissection techniques significantly improved postoperative SAQ scores including physical limitation due to angina, anginal stability, anginal frequency, treatment satisfaction, and quality of life (*P* < 0.001), no significant difference was observed between the traditional and blunt dissection techniques for SAQ. No cases of left anterior descending (LAD) injury in the blunt dissection group were observed although seven patients in the conventional group had LAD injuries.

**Conclusions:**

In our single-center experience of MB unroofing, the blunt dissection technique is a safe, effective technique that significantly reduces surgical and ventilator time and hospital stay. MB patients with severe angina who underwent the blunt dissection for surgical unroofing experienced significant improvements in anginal symptoms and quality of life six months after the surgery.

## 1. Introduction

The myocardial bridging (MB) is a congenital coronary anomaly in which the ventricular myocardium encapsulates an epicardial coronary artery, typically the left anterior descending (LAD) artery [1]. Nearly one in four people in the United States is affected by MB [2]. While a lot of them are asymptomatic, some develop angina even in the absence of significant obstructive coronary disease. MBs have often been associated with ventricular arrhythmias, acute coronary syndrome, and sudden death [3–5].

Patients with refractory angina who have failed to respond despite maximal medical therapy may be good candidates for surgical interventions. However, percutaneous coronary intervention is associated with poor outcomes in both cases due to a high rate of stent fracture and in-stent restenosis in the former [6, 7]. Therefore, surgery is an effective, albeit invasive, option for treatment for symptomatic MB patients who have failed to respond to other medical treatments. Coronary artery bypass grafting or supra-arterial myotomy also known as “unroofing” is the most common surgical option for MB. A retrospective study by Bockeria et al. which is ranked among the largest studies on bypass grafting with the left internal mammary artery to the LAD for MB has revealed extremely high rates of graft failure [8].

Myotomy involves the careful dissection of the myocardial segment overlying the epicardial coronary artery. This improves the upstream pathophysiological abnormality, thereby making it an appealing option for treatment [9]. However, studies on the outcomes of surgical procedures are scarce. Previous investigations on the outcomes of conventional unroofing have revealed that (a) the epicardium overlying the LAD, where it emerged from the myocardium and epicardial fat distal to the MB, was divided by the Beaver blade [10]; (b) tenotomy scissors were used to divide the overlying epicardial fat and MB as soon as they entered the surgical plane immediately anterior to the coronary artery, moving from distal to proximal direction until the entire myocardial bridge tissue had been removed [9]. Ventricular wall perforation (most frequently in the right ventricle with a LAD-MB and deep endomyocardial course), artery perforation, ventricular aneurysm formation, incomplete unroofing, and postoperative bleeding are all possible complications during and/or after the surgery of MB. In this study, the surgical unroofing with the blunt dissection technique in adult patients with MB has been described and evaluated using the Seattle Angina Questionnaire (SAQ) and symptomatic outcome.

## 2. Patients and Methods

### 2.1. Study Design

This retrospective study included forty-five adult patients (age ≥18 years) who underwent MB unroofing at our institution between January 1st, 2016, and December 31st, 2021. This study was approved by the Medical Ethics Review Board at the Lanzhou University Second Hospital. MBs were diagnosed using a rigorous protocol, as previously described by Boyd et al. [9]. In brief, an initial suspicion of MB was derived from stress echocardiography revealing septal buckling with apical sparing and/or from coronary computed tomographic (CT) angiography showing anatomic evidence of an MB. All patients over 18 years and diagnosed with MB were included in our study. Predefined exclusion criteria included unstable angina pectoris, uncontrolled systemic hypertension, valvular heart disease, left ventricular ejection fraction <40%, and/or significant endocrine, hepatic, renal, or inflammatory disease. An invasive angiography was performed along with coronary angiography to confirm and characterize the MB. Before surgery, coronary angiography showed systolic compression more than or equal to 70% in all patients with LAD‐MB. Patients with hemodynamically significant MB who failed maximally tolerated medical therapy were referred for surgical MB unroofing.

Adverse events (AEs) and major adverse cardiac or cerebrovascular events (MACCEs) were followed during the first 6 months postsurgery. MACCE was defined as a composite of death of any cause, myocardial infarction, stroke, or repeat revascularization. Adverse events comprised left anterior descending injury, blood transfusion, postoperative atrial fibrillation, surgical site infection, or recurrence of angina.

### 2.2. Seattle Angina Questionnaire

The Seattle Angina Questionnaire (SAQ) assesses physical limitations, angina stability, angina frequency, treatment satisfaction, and quality of life of the MB patients [11]. The SAQ scale ranges from 0 (severe symptoms) to 100 (no symptoms) for each category, with a difference in score of more than 10 being considered clinically significant. The patients completed SAQ preoperatively and again 6 months postoperatively.

### 2.3. Surgical Technique

**Figure 1:**
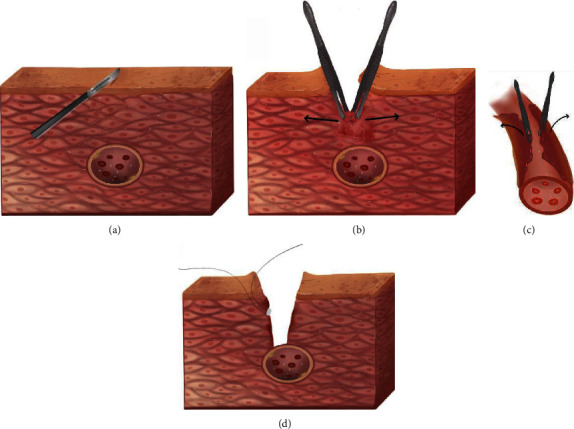
Schematic diagram of surgical unroofing using the blunt dissection technique. (a) The epicardium overlying the LAD was divided by a Beaver blade; (b) all free coronary artery procedures were carried out using the blunt dissection method; (c) the blunt dissection method was used to divide the overlying tunica externa of MB; (d) a combination of Bovie cautery, clips, and suture ligation was used to control epicardial venous bleeding.

Although there has been an evolution in our institution's surgical technique over the last five years, yet the critical portion of the procedure, i.e., complete unroofing of the entire MB has remained unchanged. The procedures involve a median sternotomy and an off-pump on a beating heart. The first 26 patients were done using traditional surgical techniques (conventional group). A Beaver blade was used to divide the epicardium overlying the LAD where it emerged from the myocardium and the epicardial fat distal to the MB. Upon entering the surgical plane, immediately anterior to the coronary artery, tenotomy scissors were used to divide the overlying epicardial fat and MB, moving in a distal to a proximal direction in 1 mm to 2 mm increments until the entire myocardial bridge tissue had been released. A combination of Bovie cautery, clips, and suture ligation was used to control epicardial venous bleeding. A pledget 4–0 polypropylene suture was placed in a horizontal mattress fashion to control an inadvertent entry into the right ventricle, a rare occurrence, similar to the technique described to control a stab wound to the heart close to a coronary artery. The remaining 19 patients underwent surgery involving a blunt dissection technique for surgical unroofing (blunt dissection group). Apart from the procedures mentioned above, all free coronary artery procedures are performed by the blunt dissection method as shown in Figure 1. The operating surgeon and referring cardiologist obtained CT images to verify that all myocardial fibers crossing the LAD had been divided and also to measure the length of the unroofed artery.

### 2.4. Statistical Analysis

The Kolmogorov–Smirnov test was used to determine the data's normality, which was further confirmed using histogram plots. For parametric data, the results are expressed as mean ± standard deviation, and nonparametric data have been expressed as the median (interquartile range). The differences between categorical variables were analyzed using *χ*^2^ tests. In case of differences between the groups of continuous variables, Student's *t*-test or Mann–Whitney test was performed. A *P* value <0.05 on both sides was considered significant. IBM SPSS Statistics 22.0 was used for statistical analysis (IBM Corporation, Armonk, NY).

## 3. Results

No significant baseline differences were observed between the two groups.  Table 1 lists the demographic characteristics of the cohort, as well as the incidence of the traditional coronary heart disease risk factors in this population. The majority of them were male patients between the age of 50 to 60 years who had undergone previous cardiac surgeries, and among them, two patients had previously undergone percutaneous coronary intervention.  Table 2 lists the surgical and postoperative characteristics of the patients. No instances of LAD injury in the blunt dissection group were observed. However, 7 patients in the conventional group had LAD injuries. MB patients in the conventional group had longer unroofed times than those in the blunt dissection group (18.91 ± 3.78 vs. 14.69 ± 1.40 min, *P* = 0.001). Postoperatively, MB patients in the conventional group had a longer ventilator time and a longer total hospital stay than those operated on using the blunt dissection technique (24.62 ± 3.09 vs. 16.26 ± 3.35 hours, *P* < 0.001; 12.89 ± 2.91 vs. 8.74 ± 1.24 days, *P* < 0.001; respectively). No blood transfusion, main adverse cardiovascular and cerebrovascular events, or surgical site infection was reported in the blunt dissection group. Although, not significantly different, the conventional group had more adverse events including blood transfusion (*n* = 4), MACCE (*n* = 3), postoperative atrial fibrillation (*n* = 4), surgical site infection (*n* = 1), and recurrence angina (*n* = 4). Two cases of stroke and one case of myocardial infarction were reported in the conventional group (Table 2).

### 3.1. Seattle Angina Questionnaire Scores

Most patients in both the groups demonstrated significant improvements across all SAQ metrics including physical limitation due to angina, anginal stability, anginal frequency, treatment satisfaction, and quality of life (*P* < 0.001) at 6 months post-MB unroofing (Table 3 lists the Seattle Angina Questionnaire Scores before and after unroofing in the conventional group and Table 4lists the Seattle Angina Questionnaire Scores before and after unroofing in the blunt dissection group). Furthermore, there were no significant differences in preoperative and postoperative SAQ scores between conventional and blunt dissection groups (Table 5 lists the Seattle Angina Questionnaire Scores before surgery between the conventional and blunt dissection groups and Table 6lists the Seattle Angina Questionnaire Scores postoperation between the conventional and blunt dissection group).

### 3.2. Comment

The results of our study demonstrated that blunt dissection can be used safely to unroof the MB in selected patients, providing a more durable option for treatment. Supra-arterial myotomy, or surgical unroofing, remains the procedure of choice for hemodynamically significant and symptomatic LAD-MBs despite maximally tolerated medical management due to low risk and its symptomatic benefits [12]. Significant symptomatic improvements were observed across all dimensions of the SAQ postsurgical unroofing of the patients. Not only was the improvement, noteworthy, but also the severity of their symptoms and poor quality of life in these patients at baseline were striking. Despite maximal tolerated medical therapy, a cohort of patients with symptomatic MBs has been struggling with anginal symptoms. Surgical unroofing of LAD-MBs directly addresses the pathology, unlike other treatment strategies that were originally intended to address another disease.

Myocardial bridging unroofing can be safely performed by an experienced surgeon either on-pump or off-pump without any major morbidity or mortality. The tenotomy technique potentially reduces the risk of coronary artery injuries and inadvertent entry into either ventricular cavity [13, 14]. Performing a complete unroofing is of paramount importance and imaging studies as a means of diagnostic evaluation provide meaningful resources for surgical planning and execution, thereby minimizing the risks of surgical complications even further [15]. All MB patients in this study were operated on off-pump. Thus, it is possible to achieve stable exposure using certain techniques and instruments.

With the help of a cardiac surface fixator, the traditional myocardial bridging release operation involves the use of a small round knife to break the myocardial bridge tissue from superficial to deep along the abnormal coronary artery until the complete exposure of the coronary artery is achieved. However, operating on the beating heart can easily cause right ventricular rupture or injury of the coronary artery, resulting in complications like bleeding, difficulty in hemostasis, incomplete release, long-term scar formation, and symptom recurrence. It is therefore a skill that needs to be mastered by surgeons who intend to operate on a beating heart. Wan and Wu reported an investigation in which 7 patients underwent traditional myocardial bridging unroofing, out of which 1 case of right ventricular perforation and its repair was identified [16]. Huang et al. also reported a similar observation in which 1 out of 3 patients undergoing traditional myocardial bridging unroofing had right ventricular perforation and was converted to coronary artery bypass grafting under cardiopulmonary bypass [17]. Our institution uses a “blunt dissection method” in which a circular knife is used to create a longitudinal cut to open the superficial criminal myocardium and then bluntly tear open the criminal myocardium near the coronary artery with the help of fine surgical tweezers. Some of the major advantages of this method include shorter operation time, lower bleeding, lower injury to the coronary artery, and easy to grasp.

### 3.3. Study Limitations

The absence of a large number of patients remains the most significant limitation of this investigation. Furthermore, there is no invasive hemodynamic data after the surgical procedure as it exposes the patient to unnecessary risk for marginal to no clinical benefit. Unless clinical situations arise justifying postoperative invasive evaluation or computational methods are developed providing postsurgical hemodynamic data, validated symptom analysis methods offer the best opportunity to measure the results of the procedure. Finally, the follow-up with the SAQ was done only for the first 6 months postsurgery, and long-term data are lacking.

## 4. Conclusion

In summary, the blunt dissection technique of MB unroofing is, therefore, found to be a safe and effective technique that significantly reduces surgical and ventilator time and hospital stay. Significant improvements in anginal symptoms and quality of life six months postsurgery were reported in MB patients with severe angina who underwent the blunt dissection.

## Figures and Tables

**Table 1:** Demographic and baseline characteristics in the study population including risk factors of the traditional coronary heart disease.

Characteristics	Conventional group (*N* = 26)	Blunt dissection group (*N* = 19)	*P* value
Age ± SD (years)	57 ± 7	54 ± 7	0.079
Male, *n* (%)	15 (58%)	12 (63%)	0.712
Body mass index ± SD (kg/m^2^)	25 ± 2	26 ± 3	0.569
Coronary artery disease, *n* (%)	12 (46%)	9 (47%)	0.936
Prior myocardial infarction, *n* (%)	5 (19%)	2 (11%)	0.426
Prior cardiac operation, *n* (%)	2 (8%)	0	0.501
Prior sternotomy, *n* (%)	1 (4%)	0	0.990
Prior CABG to LAD, *n* (%)	1 (4%)	0	0.990
Prior PCI, *n* (%)	2 (8%)	1 (5%)	0.990
Ejection fraction ± SD	57.50 ± 5.06	59.84 ± 4.20	0.108
Diabetes mellitus, *n* (%)	2 (8%)	3 (16%)	0.636
Hypertension, *n* (%)	5 (19%)	4 (21%)	0.880
Dyslipidemia, *n* (%)	8 (31%)	5 (26%)	0.745
Prior stroke or transient ischemic attack, *n* (%)	3 (12%)	1 (5%)	0.841
Chronic lung disease, *n* (%)	5 (19%)	3 (16%)	0.990
Prior atrial fibrillation, *n* (%)	3 (12%)	1 (5%)	0.841
MB total length ± SD (cm)	2.93 ± 0.86	2.77 ± 0.74	0.513
MB maximum thickness ± SD (cm)	1.70 ± 0.39	1.51 ± 0.50	0.126

CABG, coronary artery bypass grafting; LAD, left anterior descending; PCI, percutaneous coronary intervention; MB, myocardial bridging. Continuous variables are expressed as mean +SD, or the median (interquartile range), and categorical variables are expressed as number (%).

**Table 2:** Surgical and postoperative characteristics of the patients in both the groups.

Data	Conventional group (*N* = 26)	Blunt dissection group (*N* = 19)	*P* value
Unroofed times ± SD (min)	18.91 ± 3.78	14.69 ± 1.40	0.001
Length unroofed ± SD (cm)	2.64 ± 0.78	2.50 ± 0.68	0.555
Ventilator time ± SD (h)	24.62 ± 3.09	16.26 ± 3.35	<0.001
Hospital stay ± SD (d)	12.89 ± 2.91	8.74 ± 1.24	<0.001
LAD injury	7 (27%)	0	0.082
Blood transfusion, *n* (%)	4 (15%)	0	0.126
MACCE, *n* (%)	3 (12%)	0	0.252
Stroke, *n* (%)	2 (8%)	0	0.501
Myocardial infarction, *n* (%)	1 (4%)	0	0.990
Postoperative atrial fibrillation, *n* (%)	4 (15%)	2 (11%)	0.976
Surgical site infection, *n* (%)	1 (4%)	0	0.990
Recurrence angina, *n* (%)	4 (15%)	2 (11%)	0.976

LAD; left anterior descending; MACCE, major adverse cardiac or cerebrovascular event. Continuous variables are expressed as mean ± SD, or the median (interquartile range), and categorical variables are expressed as number (%).

**Table 3:** Seattle Angina Questionnaire Scores before and after unroofing in the conventional group.

Categories	Preoperative (*n* = 26)	Postoperative (*n* = 26)	*P* value
Physical limitation due to angina	71.6 (64.4–77.8)	88.6 (80–95.6)	<0.001
Anginal stability	17.3 (0–50)	78.8 (50–100)	<0.001
Anginal frequency	59.6 (50–70)	85.7 (70–100)	<0.001
Treatment satisfaction	52.0 (41.2–64.7)	85.3 (70.6–94.1)	<0.001
Quality of life	48.5 (38.5–61.5)	81.3 (76.9–100)	<0.001

Data were expressed as the median (interquartile range).

**Table 4:** Seattle angina questionnaire scores before and after unroofing in the blunt dissection group.

Category	Preoperative (*n* = 19)	Postoperative (*n* = 19)	*P* value
Physical limitation due to angina	69.4 (62.2–77.8)	86.9 (80–91.1)	<0.001
Anginal stability	11.8 (0–50)	73.7 (50–100)	<0.001
Anginal frequency	56.8 (40–70)	86.3 (70–100)	<0.001
Treatment satisfaction	50.7 (35.3–64.7)	80.2 (70.6–88.2)	<0.001
Quality of life	45.7 (30.8–53.8)	84.6 (76.9–100)	<0.001

Data was expressed as the median (interquartile range).

**Table 5:** Seattle Angina Questionnaire Scores before unroofing operation in the two groups.

Categories	Conventional group (*N* = 26)	Blunt dissection group (*N* = 19)	*P* value
Physical limitation due to angina	71.6 (64.4–77.8)	69.4 (62.2–77.8)	0.100
Anginal stability	17.3 (0–50)	11.8 (0–50)	0.355
Anginal frequency	59.6 (50–70)	56.8 (40–70)	0.529
Treatment satisfaction	52.0 (41.2–64.7)	50.7 (35.3–64.7)	0.667
Quality of life	48.5 (38.5–61.5)	45.7 (30.8–53.8)	0.398

Data were expressed as the median (interquartile range).

**Table 6:** Seattle Angina Questionnaire Scores after unroofing operation in the two groups.

Categories	Conventional group (*N* = 26)	Blunt dissection group (*N* = 19)	*P* value
Physical limitation due to angina	88.6 (80–95.6)	86.9 (80–91.1)	0.262
Anginal stability	78.8 (50–100)	73.7 (50–100)	0.219
Anginal frequency	85.7 (70–100)	86.3 (70–100)	0.691
Treatment satisfaction	85.3 (70.6–94.1)	80.2 (70.6–88.2)	0.206
Quality of life	81.3 (76.9–100)	84.6 (76.9–100)	0.152

Data were expressed as the median (interquartile range).

## Data Availability

All data used during the study are included within the submitted article.

## Additional Points

*Institutional Review Board Approval or Waiver*. All adult patients (age ≥18 years) who underwent MB unroofing at our institution between January 1st, 2016, and December 31st, 2021, were included in this retrospective study (*N* = 45), which was approved by the Medical Ethics Review Board at the Lanzhou University Second Hospital.
